# Clinical and physiological advances in sedentary behavior research

**DOI:** 10.3389/fphys.2024.1348122

**Published:** 2024-03-14

**Authors:** Ilkka Heinonen

**Affiliations:** Turku PET Centre, University of Turku and Turku University Hospital, Turku, Finland

**Keywords:** sedentary behavior, physical activity, randomized controlled trials, clinical, physiology, health, cardiometabolic, humans

## Abstract

Sedentary behavior, defined as sitting with low energy expenditure, has emerged as a modifiable risk factor that affects our physiology and health. Evidence for the detrimental effects of sedentary behavior/physical inactivity on health, however, stems largely from epidemiological studies, which cannot address causalities. Acute and short-term sedentary behavior reduction interventions have been performed; however, in these studies, sitting has often been replaced by formal physical activity options, such as exercise, and long-term studies in subjects with cardiometabolic risk factors are still relatively few. We have recently conducted a long-term randomized controlled trial (RCT) to reduce daily sitting, without formal exercise, in metabolic syndrome patients, and this mini-review presents these studies with physiological aspects. The findings indicate that sedentary behavior reduction can prevent the increase in levels of many cardiometabolic risk factors after 3 months, but more intense physical activity rather than only reducing daily sitting time may be needed to further reduce the risk factor levels. At 6-month time point reduced sitting reduced fasting insulin, while successfully reducing sitting and body fat had beneficial effects also on whole-body insulin sensitivity, but other effects were relatively minor. Reduced sitting did not improve maximal aerobic fitness after 6 months, but an increase in daily steps was positively associated with an increase in fitness. However, the more the participants replaced sitting with standing, the more their maximal aerobic fitness was reduced. Overall, although the analysis of the collected data is still ongoing, our RCT findings suggest that the physiological and health effects of reduced sitting are relatively minor and that physical activities such as taking more daily walking steps are needed, which would be more beneficial and time-efficient for improving human health.

## Introduction

*Sede* means *to sit*, and sedentary behavior thus means a behavior where a person spends time lying or sitting. Energy expenditure during sitting is not much higher than that when lying but little lower than that when standing. Sedentary behavior has been associated with many detriments to health, such as cardiometabolic diseases and early mortality (Lee et al., 2012; Dempsey et al., 2020). It is often claimed to be independent of physical inactivity, and although this might be true when a person does not exercise enough, a reasonable amount of physical activity and exercise has also been shown to completely eliminate the deleterious effects of sedentary behavior (Ekelund et al., 2016; Sagelv et al., 2023). In many (epidemiological) studies, their associations have been investigated by adjusting for physical activity, but whether sedentary behavior means lack of meeting the current physical activity recommendations, which is 150 min per week of moderate-intensity physical activity or 75 min of moderate-to-vigorous-intensity activity and thus not all physical activities, is not clearly defined. Recommendations and advocates in the field, however, also often state that every movement counts and that just a little movement is better than none, and the more is better than less. We must therefore bear in mind that light-intensity physical activity is also physical activity and must be taken into account when addressing the possible detrimental effects of sedentary behavior and the possible health benefits of reduced daily sitting. Furthermore, time does not disappear, and thus it means that every action that is replaced during the day automatically means some other activity is increased.

Physical activity means any bodily movements or muscle contractions that increase energy consumption during leisure time, and therefore physical inactivity, thus lack of physical activity, is a perfect term (van der Ploeg and Hillsdon, 2017; Harris, 2023) to describe sedentary behavior, although it is often stated that they are not the same. Taken together, sedentary behavior/physical inactivity form an energy consumption continuum with physical activity where standing lies somewhere in the middle. Importantly, it is often neglected in sedentary behavior research that whether the control and adjustment of diet and energy intake contribute to health improvement. If one is not physically active, it might be difficult to eat too little to match energy consumption to energy intake and avoid energy surplus that leads to excess adiposity, which is also a common feature of sedentary behavior. Research shows that the control of energy intake reduces detriments to health , although physical inactivity *per se* is still detrimental physiologically (Stephens et al., 2011). Furthermore, when dietary energy intake as well as the quality of diet is controlled in addition to genetic predisposition to body adiposity, sedentary behavior is an independent predictor of body adiposity (Heinonen et al., 2013). Nevertheless, a food diary should always be included in sedentary behavior research, and results should be adjusted by changes in diet if they occur.

However, the main issue still in the field of sedentary behavior research is that the evidence for its detrimental role still stems largely from epidemiological studies, which cannot adequately address the causality of the findings. Therefore, more clinically and physiologically oriented intervention studies are urgently needed to investigate the idea that whether sedentary behavior reduction improves health. To address this issue, a randomized controlled trial (RCT) was conducted, in which subjects were randomized either into the intervention or control group. Whereas participants in the control group should continue their normal lifestyle without any changes, participants in the intervention group should reduce their time spent sitting. This study further investigated whether this reduced sitting translates into improved health, and if so, through which mechanisms.

## Clinical and physiological intervention studies

In the year 2023, a seminal review on the current knowledge of physiology and the background of sedentary behavior research was published (Pinto et al., 2023). According to the reviewed evidence, evidence for sedentary behavior reduction interventions to date is mostly received from acute and fairly short-term interventional clinical and physiological studies. We have therefore conducted a 6-month RCT to study the clinical and physiological aspects of long-term sedentary behavior reduction as RCTs are generally considered the gold standard for providing the best and most trusted medical evidence to address the causality of the findings.

As the evidence shows that sedentary behavior reduction in a fairly lean and fit subject does not provide much physiological and clinical benefits (Dempsey et al., 2016), we investigated metabolic syndrome patients that had an high incidence of cardiometabolic risk factors and showed that the intervention has the potential to improve their health. The inclusion criteria were age 40–65 years; physical inactivity (<120 min/week of self-reported moderate-to-vigorous physical activity, sedentary time ≥10 h/day or ≥60% of accelerometer wear time/day during screening); body mass index (BMI) 25–40 kg m^−2^; blood pressure <160/100 mmHg; fasting glucose <7.0 mmol L^−1^; and fulfillment of metabolic syndrome criteria including at least three of the following: waist circumference ≥94 cm for men or ≥80 cm for women, triglycerides ≥1.7 mmol L^−1^, HDL <1.0 mmol L^−1^ for men or <1.3 mmol L^−1^ for women or on lipid medication, systolic blood pressure ≥130 mmHg and/or diastolic blood pressure ≥85 mmHg, or fasting glucose ≥5.6 mmol L^−1^. The exclusion criteria were a previous cardiac event; diagnosed with diabetes; excessive alcohol consumption (according to national guidelines); use of narcotics, cigarette, or snuff tobacco; depressive or bipolar disorder; and any chronic disease or condition that could endanger participant’s safety or hamper study procedures or interfere with the interpretation of results.

We first determined their baseline sedentary behavior, time spent standing, and physical activity levels during a 1-month screening phase using accelerometers with 6-s collection frequency for a whole day. As the health risks were shown to increase more clearly after 10-h sitting per day, we chose to include an intervention for those people with metabolic syndrome who sit for more than 10 h per day (or 60% of the accelerometer wear time as wear time also affects the accumulation of hours). The reference value for the daily sitting time of every participant was determined based on this 1-month baseline measurement. The intervention aimed at reducing daily sitting time by 1-h per day compared to their individual sitting time, whereas the control group was instructed to continue their normal lifestyle. Daily sitting was instructed, encouraged, and guided to be reduced by increasing standing and light-intensity physical activities such as taking more walking steps during their workday and not by increasing formal exercise. We could not, however, naturally exclude any of the participants who started to reduce their daily sitting by brisk walking sessions, for instance, as they found them to be the easiest way to change their habits. All activities were recorded using accelerometers with 6-s collection frequency every day for a 6-month period, and all participants could follow their daily sitting, standing, and physical activity accumulation using an application in their smart phones. Dietary factors were recorded at baseline and once during the 6-month intervention using a 4-day food diary (including one weekend day), but the participants were instructed not to change their normal dietary habits.

**FIGURE 1:**
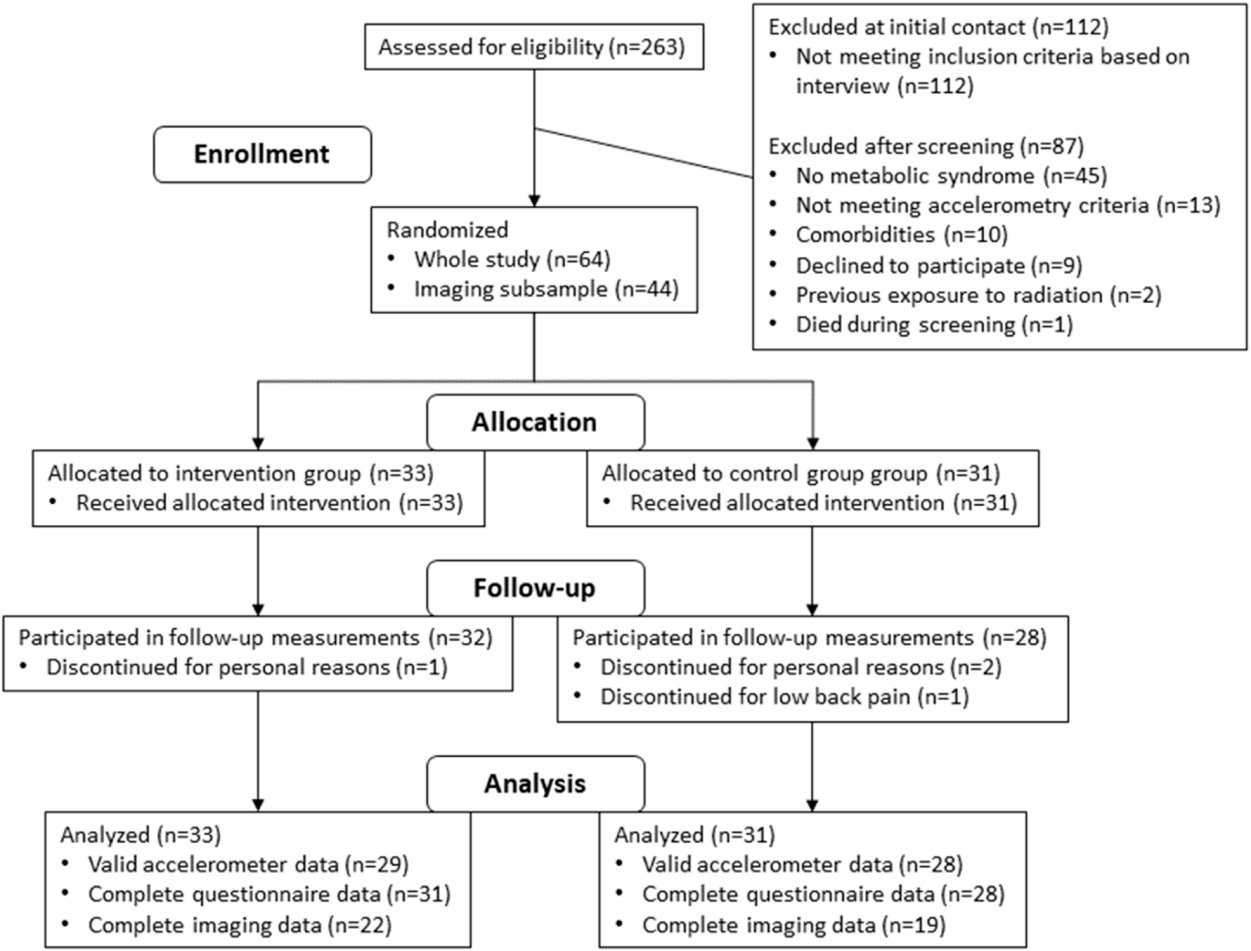
Study flow diagram of our RCT investigating the physiological and clinical benefits of reduced daily sitting in metabolic syndrome patients during a 6-month intervention.

**FIGURE 2:**
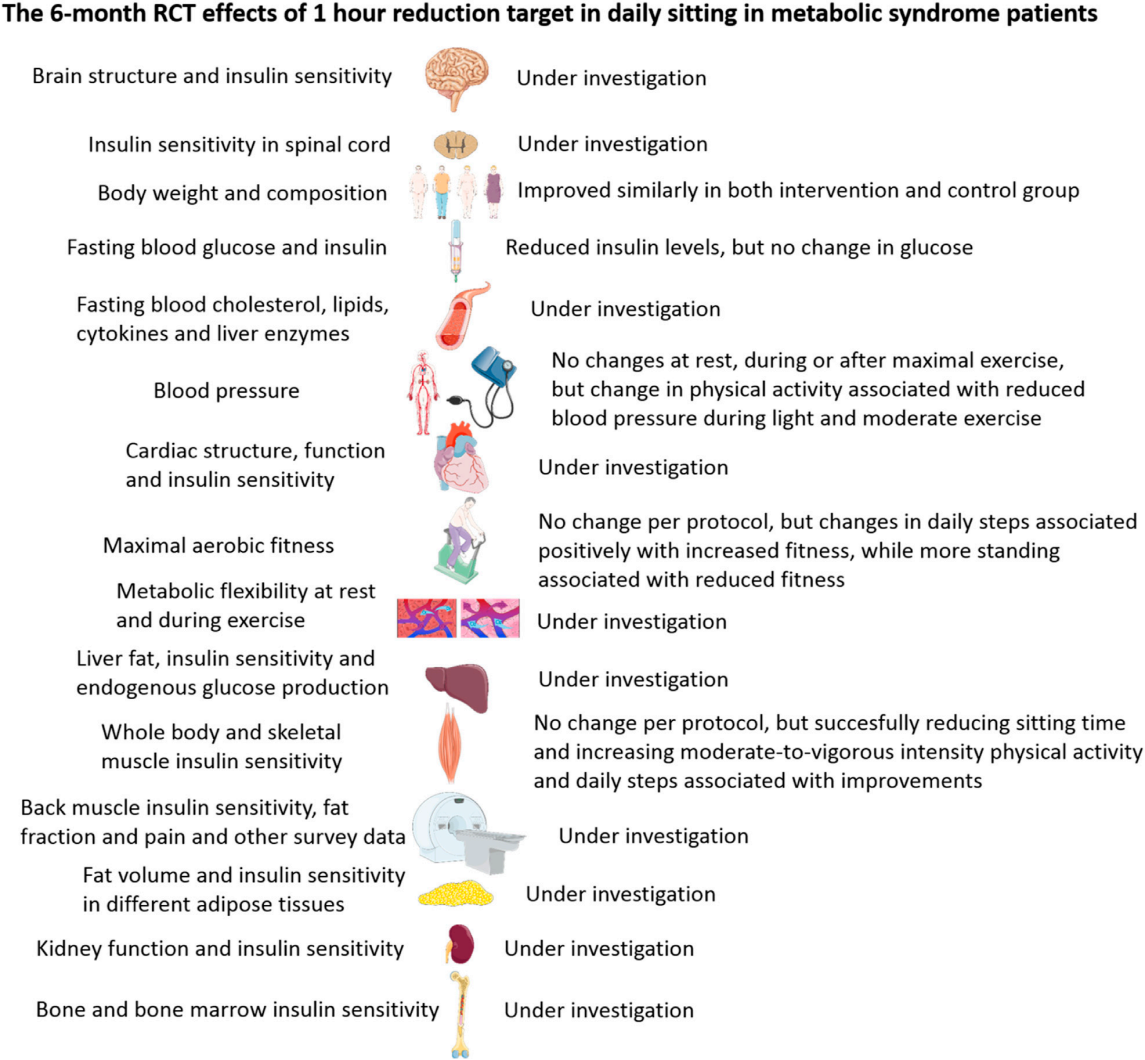
Summary of the results (Sjoros et al., 2023a; Sjoros et al., 2023b; Norha et al., 2023; Norha et al., 2024) and topics under investigation with regard to the 6-month randomized trial studying the clinical and physiological effects of reduced sitting in metabolic syndrome patients.

The study flow diagram is presented in Figure 1. Although the intervention has been completed and findings of one 3-month study and few 6-month studies have been published (Sjoros et al., 2023a; Sjoros et al., 2023b; Norha et al., 2023; Norha et al., 2024), large part of the 6-month intervention results are still under analysis. These include, but not limited to, insulin sensitivity and other anatomical and physiological research on the brain, liver, heart, adipose tissue, bone and bone marrow, and spinal cord, as well as metabolic flexibility (Figure 2).

Clinical and physiological findings from the screening phase (Figure 1) show that both sedentary behavior and physical activity are associated with numerous cardiometabolic risk factors in overweight and obese adults (Sjoros et al., 2020). However, the duration of accelerometer data collection also affects markers of insulin sensitivity and most likely, the longer the duration, the better it describes the actual behavior of an individual and provides more reliable results (Sjoros et al., 2021). Furthermore, in these studies, we commonly found that body adiposity rather than daily sitting time or physical activity is associated (detrimentally) with physiological and health measures, such as increased hemoglobin and white blood cell count (Koivula et al., 2022) and changes in liver enzymes (Laine et al., 2021). In addition and in line with the major influence of body adiposity *per se*, our pre-intervention baseline measurements have elucidated that body adiposity is also a major determinant of the association between maximal aerobic fitness and cardiometabolic health in metabolic syndrome patients (Haapala et al., 2022), and obesity and daily protein intake rather than fitness, daily sitting time, or physical activity are associated with liver fat accumulation (Laine et al., 2022). Body adiposity appears to be a more important marker for skeletal muscle insulin sensitivity than the duration of sitting time (Garthwaite et al., 2023) in metabolic syndrome patients, but interestingly, daily standing time shows a positive association with whole-body insulin sensitivity, as measured using the gold standard insulin clamp technique (Garthwaite et al., 2021). Finally, these cross-sectional investigations also suggested that both standing time and sitting time are associated with pain-related disability (Norha et al., 2022). Longer daily standing time, but not light- or moderate-intensity physical activity, is associated with higher levels of pain-related disability. On the other hand, a higher proportion of sedentary behavior is associated with lower levels of pain-related disability, suggesting that individuals with pain-related disability may prefer to stand, possibly to cope with pain (Norha et al., 2022).

In our 3-month sitting time reduction intervention study, we reported that reducing sedentary behavior is effective in preventing the increase in many cardiometabolic risk factors that occurred in the control group over time, but only reducing daily sitting time appears to be ineffective in reducing the levels of these cardiometabolic risk factors (Garthwaite et al., 2022). It may be that more pronounced reduction in daily sitting time and/or an increase in the amount of intensity of physical activity is needed to show reductions in cardiometabolic risk factors in metabolic syndrome patients. The participants in the intervention group reduced their daily sitting by 50 min on average by increasing not only their light-intensity physical activity but also their moderate-intensity physical activity. The participants in the intervention group also increased their daily walking steps from approximately 5,000 steps to over 8,000 steps; however, the control group also increased their daily steps, although they were instructed to maintain their normal lifestyle. This unintended effect likely influenced the results as it is harder to show clear intervention effects if the control group also changes their lifestyle habits; thus, this explains the purpose of including a control group and RCT design in intervention studies. The positive effects of sedentary behavior reduction intervention favoring the intervention group were, however, documented in fasting insulin, HOMA-insulin resistance, HbA_1c_, triglycerides, ALT liver enzyme, and resting heart rate (Garthwaite et al., 2022), indicating that reduced daily sitting is beneficial for health. Waist circumference, body fat percentage, fat mass, and systolic and diastolic blood pressure decreased slightly during the intervention with no difference between the groups, suggesting that simply enrollment into a behavior study and thus getting health checkups can provide beneficial health effects. The levels of fasting glucose, fat-free mass, total cholesterol, LDL- and HDL-cholesterol, and AST and GGT liver enzymes increased similarly in both groups, indicating that simply reducing daily sitting by 50 min per day is not effective in preventing the increase in these health risk factors. The weight or body mass index did not change in either group (Garthwaite et al., 2022). Furthermore, changes in standing time were inversely correlated with weight and body mass index changes, and changes in the number of steps/day were inversely correlated with waist circumference changes. Changes in moderate-to-vigorous-intensity physical activity were positively correlated with HDL changes, changes in weight and body mass index were positively correlated with changes in triglyceride levels and blood pressure, and changes in waist circumference were also positively correlated with changes in blood pressure. Changes in fat-free mass were inversely correlated with changes in fasting glucose levels (Garthwaite et al., 2022). Thus, it is evident that favorable changes in behavior and body composition result in many positive relationships that improve health.

The 6-month intervention findings illustrate that daily sitting time could be reduced by 40–50 min in the intervention group, while no changes occurred in the control group (Sjoros et al., 2023a; Sjoros et al., 2023b). The participants in the intervention group markedly increased their daily walking steps, but so did the control group, although not as markedly as the intervention group (Sjoros et al., 2023a; Sjoros et al., 2023b). This 6-month sitting time reduction intervention resulted in slightly decreased fasting insulin levels but had no per-protocol effects on insulin sensitivity or body adiposity. However, as the change in insulin sensitivity is associated with the changes in sedentary behavior and body mass, our main conclusion based on this study is that multifaceted interventions targeting weight loss are likely to be beneficial in improving whole-body insulin sensitivity (Sjoros et al., 2023b). Furthermore, additional analyses showed that successfully reducing daily sitting time also led to improvements in whole-body insulin sensitivity. Favorable, tissue-specific insulin sensitivity improvements can also be seen in skeletal muscles but only in postural muscles that were likely activated during the intervention (Sjoros et al., 2023a). Furthermore, simply reducing sitting time does not improve maximal aerobic fitness (Norha et al., 2023). However, the more the participants increased their daily walking steps in the 6-month period, the better their fitness improved (Norha et al., 2023). Interestingly, the more they increased their daily standing, the more the aerobic fitness was reduced (Norha et al., 2023). It appears that if sitting is replaced mostly by standing, it is not beneficial for aerobic fitness as standing does not stress the body adequately; alternatively, actual muscle movements are needed, which increases total body energy consumption more substantially and creates the demand for fitness improvement. Furthermore, sedentary behavior reduction did not affect blood pressure at rest or during exercise until maximal exhaustion and recovery from it, but changes in physical activity with the 6-month intervention was associated with reduced blood pressure during corresponding activity levels during the exercise test (Norha et al., 2024). This can be considered beneficial for health and supports the idea of replacing sitting by physical activity for beneficial cardiovascular effects. However, it remains to be investigated whether blood lipids are beneficially affected by reduction in sitting time compared to physical activity (Kujala et al., 2013).

In conclusion, although many physiological aspects are still under investigation in different tissues, it can be said that successfully reducing sedentary behavior in metabolic syndrome patients, a common feature in Western countries, can provide many health benefits and their effects appear to be fairly minor. It is evident that sedentary behavior reduction can prevent the increase in risk factors over time rather than actually reducing their levels, although fasting insulin levels were reduced by reducing daily sitting time at the 6-month time point. Although reducing daily sitting time is definitely a good initiative for everyone, physical activity and exercise would be more time-efficient (Biswas et al., 2018) and beneficial for improving health. The findings also point to the conclusion that replacing sitting by movement rather than by standing is more beneficial for fitness and health and that multifaceted interventions that also focus on diet and body weight reduction are needed to complement sitting time reduction. Finally, a 40–50 min reduction in daily sitting time may not be enough, but 1–3 h or more, preferably by a concomitant increase in physical activity rather than simply standing, is needed for clear health improvements.
